# Outcomes of a Remotely Delivered Complementary and Integrative Health Partnered Intervention to Improve Chronic Pain and Posttraumatic Stress Disorder Symptoms: Randomized Controlled Trial

**DOI:** 10.2196/57322

**Published:** 2024-10-18

**Authors:** Jolie N Haun, Christopher A Fowler, Hari H Venkatachalam, Amy C Alman, Lisa M Ballistrea, Tali Schneider, Rachel C Benzinger, Christine Melillo, Neil B Alexander, S Angel Klanchar, William A Lapcevic, Matthew J Bair, Stephanie L Taylor, Jennifer L Murphy, Dustin D French

**Affiliations:** 1 Research and Development Service James A Haley Veterans' Hospital Tampa, FL United States; 2 Division of Epidemiology Department of Internal Medicine University of Utah Salt Lake City, UT United States; 3 Department of Psychiatry and Behavioral Neurosciences University of South Florida Tampa, FL United States; 4 College of Public Health University of South Florida Tampa, FL United States; 5 Geriatric Research Education and Clinical Center VA Ann Arbor Healthcare System Ann Arbor, MI United States; 6 Division of Geriatric and Palliative Medicine Department of Internal Medicine University of Michigan Ann Arbor, MI United States; 7 Health Services Research and Development Center for Health Information and Communication Roudebush VA Medical Center Department of Veterans Affairs Indianapolis, IN United States; 8 Department of Medicine School of Medicine Indiana University Indianapolis, IN United States; 9 Regenstrief Institute Inc Indianapolis, IN United States; 10 Health Services Research and Development Center for the Study of Healthcare Innovation, Implementation, and Policy VA Greater Los Angeles Healthcare System Department of Veterans Affairs Los Angeles, CA United States; 11 Department of Medicine David Geffen School of Medicine University of California Los Angeles, CA United States; 12 Department of Health Policy and Management Fielding School of Public Health University of California Los Angeles, CA United States; 13 National Pain Management, Opioid Safety, and Prescription Drug Monitoring Program Specialty Care Program Office Veterans Health Administration Washington, DC, DC United States; 14 Health Services Research and Development Center of Innovation for Complex Chronic Healthcare Edward Hines Jr VA Hospital Department of Veterans Affairs Hines, IL United States; 15 Center for Health Services and Outcomes Research Feinberg School of Medicine Northwestern University Chicago, IL United States; 16 Departments of Ophthalmology and Medical Social Sciences Feinberg School of Medicine Northwestern University Chicago, IL United States

**Keywords:** posttraumatic stress disorder, PTSD, pain, veteran, attrition, complementary and integrative health, CIH, randomized controlled trial, chronic pain, remote intervention, dyad, mobile health

## Abstract

**Background:**

Nonpharmacological interventions for veterans are needed to help them manage chronic pain and posttraumatic stress disorder (PTSD) symptoms. Complementary and integrative health (CIH) interventions such as Mission Reconnect (MR) seek to provide veterans with the option of a partnered, self-directed intervention that teaches CIH skills remotely to support symptom management.

**Objective:**

The purpose of this study was to describe the physical, psychological, and social outcomes of a self-directed mobile- and web-based CIH intervention for veterans with comorbid chronic pain and PTSD and their partners and qualitatively examine their MR user experience.

**Methods:**

A sample of veteran-partner dyads (n=364) were recruited to participate in a mixed methods multisite waitlist control randomized controlled trial to measure physical, psychological, and social outcomes, with pain as the primary outcome and PTSD, depression, stress, sleep, quality of life, and relationships as secondary outcomes. Linear mixed models were constructed for primary and secondary patient-reported outcomes. The quantitative analysis was triangulated using qualitative interviews from a subsample of dyads (n=35) to examine participants’ perceptions of their program experience.

**Results:**

Dyads were randomized to 2 groups: intervention (MR; 140/364, 38.5%) and waitlist control (136/364, 37.4%). No significant change was observed in overall pain, sleep, PTSD, quality of life, relationship satisfaction, overall self-compassion, or compassion for others. A significant reduction in pain interference in mood (*P*=.008) and sleep (*P*=.008) was observed among the veteran MR group that was not observed in the waitlist control group. We also observed a positive effect of the MR intervention on a reduction in negative affect associated with pain (*P*=.049), but this effect did not exceed the adjusted significance threshold (*P*=.01). Significant improvements were also observed for partners in the affection (*P=*.007) and conflict (*P=*.001) subdomains of the consensus and satisfaction domains. In contrast to quantitative results, qualitative data indicated that intervention impacts included improved sleep and reduced pain, anxiety, and stress and, in contrast to the survey data, overall improvement in PTSD symptoms and social relationships. Participants’ overall impressions of MR highlight usability and navigation, perceptions on packaging and content, and barriers to and facilitators of MR use.

**Conclusions:**

Adjunctive CIH-based modalities can be delivered using web and mobile apps but should be developed and tailored using established best practices. MR may be beneficial for veterans with pain and PTSD and their partners. Further pragmatic trials and implementation efforts are warranted.

**Trial Registration:**

ClinicalTrials.gov NCT03593772; https://clinicaltrials.gov/study/NCT03593772

**International Registered Report Identifier (IRRID):**

RR2-10.2196/13666

## Introduction

### Background

Chronic pain is an ongoing, debilitating morbidity that impacts an estimated 50 million Americans, roughly 20% of the US population [1]. Compared to the general population, veterans disproportionally experience chronic pain overall (29.1% vs 19.5%) [1], and their incidence of severe chronic pain is higher (9.7% vs 6.4%) [2]. In veterans, chronic pain commonly co-occurs with posttraumatic stress disorder (PTSD), which can complicate therapy options and exacerbate symptom severity [3-5]. In veteran clinical pain populations, the estimated PTSD prevalence is 11.7% [6]. Co-occurrence of chronic pain and PTSD is associated with poor outcomes, such as elevated PTSD symptoms, pain intensity, sleep issues, pain catastrophizing beliefs, and psychological health outcomes [7-9].

Driven by need for nonpharmacological interventions, evidence, and veteran demand, Subtitle C of the 2016 Comprehensive Addiction and Recovery Act mandated expansion of research, education, and delivery of complementary and integrative health (CIH) for veterans receiving pain and mental health services [10]. Following the Comprehensive Addiction and Recovery Act, Veterans Health Administration (VHA) Directive 1137 [11] supported transformation to a whole health system of care to support proactive delivery of evidence-based CIH modalities to address veterans’ health and wellness needs while also creating alternatives for nonpharmacological options in a national opioid epidemic [11]. In this transformation to whole health, 8 evidence-based CIH therapies were identified and included at the Department of Veterans Affairs (VA) facilities as medical benefits—acupuncture, meditation, tai chi, yoga, massage therapy, guided imagery, biofeedback, and clinical hypnosis [12-18]. These CIH modalities have demonstrated significant impacts in military and veteran populations in managing pain intensity and physical functioning [19].

While whole health and CIH services provide an evidence-based biopsychosocial integrative approach to pain management [20], examining the use of CIH-based mobile apps and resources to complement in-person clinical care [21] is warranted. Mission Reconnect (MR) is a remotely delivered, mobile and web-based CIH-based didactic self-care intervention (ie, education, massage therapy, meditation, and positive psychology) that leverages social relationships between veterans and their partners and has demonstrated improvements in pain and PTSD measures among veterans and service members in a powered randomized controlled trial conducted in a community-based setting. The MR intervention has been further detailed in a previous publication [9].

Massage therapy, the primary partnered CIH component of MR, has been examined extensively as a nonpharmacologic approach to pain management [22-26] in diverse populations. More recently, the VA has supported efforts to examine the potential effects of massage therapy [27,28], with results indicating potential to support pain management. Massage therapy and mindfulness have shown low- to moderate-quality evidence, indicating improvement in low back pain, short term (ie, immediate effects after the treatment) [24,29-37], cancer pain [25], and surgical pain [26]. For other areas of pain (eg, shoulder and neck), the evidence is conflicting, with primarily low-quality, short-term studies [30,35,38]. Although the field has shown interest in the use of massage therapy with other CIH modalities to manage PTSD-related symptoms, currently, research is limited [39-45]. Although a few studies have identified the potential benefit of the use of massage therapy and CIH-related modalities [39-41] to treat PTSD, others have indicated insignificant effects [42,44,45]. MR also provides “movement” exercises for veterans and their partners; this inclusion is based on the documented benefit of CIH such as yoga and tai chi for managing pain [15,46-50], including short-term and long-term impacts on pain in diverse populations [51].

Finally, MR also includes a mindfulness-based component, which has been associated with short-term effects on pain intensity and physical functioning [33,52-55]. Several reviews have indicated that mindfulness-based interventions have significant effects on neck pain [46] and low back pain specifically [48,56-61]; meta-analytic results indicate large effect sizes for improving pain intensity, unpleasantness, and interference [62,63]. In contrast, a recent review in a veteran population indicated that mindfulness-based stress reduction therapies had significant impacts on measures of PTSD, depression, general psychological symptoms, quality of life (QOL) and functioning, and mindfulness, but not on physical health and did not sustain effects at follow-up [64]. A separate meta-analysis examining meditation for improving depression symptoms in participants with chronic pain indicated small effect sizes and poor study quality [65]. There is evidence suggesting that mind-body exercises (eg, yoga, tai chi, and qigong) have significant impacts on PTSD [66], depressive, and anxiety symptoms [67-69]. In general, mindfulness-based (eg, meditation) trials have demonstrated significant improvements in PTSD symptoms [70-76].

Specific to the veteran population, as early as 2011, a VA evidence review indicated that a review of studies reported short-term effects of CIH interventions on PTSD symptoms [77]. Meditation interventions have been shown to have small– to medium–effect size improvements for alleviating military-related PTSD symptoms [78]. Reviews of the literature provide evidence of significant effects using meditation-based therapy for managing PTSD symptoms in military and veteran populations [78-80]. Although the evidence is compelling, more work demonstrating the effects of CIH for managing PTSD-related symptoms using standardized protocols and high-quality reporting are warranted.

As research is needed to examine the use of CIH modalities to manage pain and PTSD in clinical populations, examination of the use of mobile and web-based technology to deliver CIH interventions is also warranted. In a scoping review of the literature examining the use of mobile and web-based resources to deliver CIH, of the 330 articles included, only 2 (excluding previous MR publications) included relevant partnered interventions [81]. A previous feasibility study of caregiver-provided massage therapy found statistically significant improvements in veteran pain, stress, and fatigue [82]. Another pilot partnered intervention using remotely telephone-delivered symptom management found improvement in pain interference and psychological distress for patient-caregiver dyads that had greater assertive communication but, surprisingly, increased pain and fatigue interference for dyads with greater mindfulness practice [83]. We are not aware of any publications on completed remotely delivered partnered interventions since our published scoping review; a search of the literature revealed only 2 protocol papers [84,85].

Specific to MR, a previous community-based pilot study demonstrated that MR use was associated with pretest-posttest improvements in pain intensity and PTSD symptoms among a sample of postdeployment service members and a consenting partner of their choosing (97.7% spouses or life partners) [86]. A randomized controlled trial of community-based post-9/11 combat veterans and their self-selected partners (94.4% spouses or life partners) found that pain intensity and PTSD symptoms improved when MR was used alone or as an adjunct to a relationship enhancement intervention compared to a waitlist control group [87]. Of note, despite PTSD symptom scores often exceeding clinically recommended cutoffs for diagnostic screening in the observed samples, formal chronic pain and PTSD diagnoses were not used as inclusion criteria in either study. Given the demonstrated efficacy of MR in reducing pain intensity and PTSD symptoms, the purpose of this study was to examine the effects of MR among a sample of veterans diagnosed with co-occurring chronic pain and PTSD within the VA system.

### Objectives

This paper presents findings from a 4-year mixed methods randomized waitlist-controlled trial aimed to test MR for chronic pain and PTSD symptom management [9]. The aims of this research were to (1) determine MR effectiveness for physical (pain and sleep), PTSD (intrusion, arousal, avoidance, and numbing), and psychological (depression, stress, and anxiety) symptoms and global health (QOL); (2) determine MR effectiveness for social (relationship satisfaction and compassion for the self or others) outcomes among veterans and their partners; and (3) describe the veteran- and partner-perceived value of MR in a subsample of dyads using qualitative interviews to explore perceived outcomes and identify recommendations for program improvement.

## Methods

### Ethical Considerations

Study procedures and all research activities were reviewed for human participants protection and approved (Pro00035440) by the institutional review boards from the universities of South Florida, Michigan, and Washington. All participants consented before taking part in the randomized controlled trial. To incentivize participation, veterans were compensated with US $20 for each of the 4 assessments and US $5 per week for providing MR use and pain rating data. Interview participants received an additional US $20, with a total compensation of up to US $140. The methodology, consent and recruitment process, and baseline attrition have been described in detail elsewhere [9,88]. The study’s methods and results have been reported in accordance with the CONSORT-EHEALTH (Consolidated Standards of Reporting Trials of Electronic and Mobile Health Applications and Online Telehealth) guidelines (Multimedia Appendix 1) [89]. Data were deidentified to protect participants’ identities. A plan describing data management and access was developed to outline the applicable data management and access details for the project.

### Design

A randomized controlled trial with a mixed methods design was used in which veterans and their partners were randomly assigned to the intervention (MR) and waitlist control arms. Repeated measurements of patient-reported outcomes were assessed within participants at weeks 1 to 8 and, finally, 16 weeks after assignment. Qualitative interviews were conducted 2 months after intervention completion. Including qualitative data to complement the quantitative findings contributed to a deeper understanding of participant experiences with the intervention. Intention-to-treat principles guided all quantitative and qualitative analyses.

### Sample

Veteran-partner dyads (n=364) were recruited from 3 urban VA medical centers located in the southeastern, midwestern, and northwestern regions of the continental United States. Partners were family members, friends, and significant others chosen by the veteran to participate in the MR program and study. Veterans were recruited and consented based on a confirmed comorbid diagnosis of PTSD and chronic pain. Participants were required to be aged ≥18 years, English speaking, and capable of performing MR intervention practices and fulfilling the technology requirements. Additional eligibility requirements were absence of reported aggression or violence within the participant dyad, no history of moderate to severe traumatic brain injury (TBI), no sensory or physical dysfunction that may interfere with study activities (ie, cognitive, hearing, and vision), no recent or current diagnosis of or inpatient treatment for psychotic or substance use disorder, and no technology barriers interfering with MR and Qualtrics (Qualtrics International Inc) [90] survey access (eg, lack of access to internet or email) [9].

The number of dyads was selected based on having adequate statistical power on the pain outcome measure to detect a small to medium effect size (Cohen *d*=0.38) given an anticipated 20% attrition rate. However, the observed attrition rate was roughly 33%, and the number of dyads recruited increased from the original target of 228 to a total of 364 dyads [88]. Participants in the qualitative inquiry comprised a subsample of 35 dyads that were recruited from the intervention arm of the parent MR study. Participants agreed to be interviewed during the consenting process. Purposive sampling methodology was applied to select participants who self-reported use of the MR website and completion of study activities within the highest and lowest terciles of use. As the number of individuals who did not respond to use questions increased, we added a randomization option to ensure that we had enough participants for data saturation.

### Materials

#### Screening Measures

A 16-item structured interview questionnaire was used to assess eligibility criteria (see the previous section) and randomization stratum (site, concurrent PTSD, and chronic pain treatment) for veteran-partner dyads. All 16 interview items were binary (*yes* or *no*) [9,88]. The Ohio State University TBI Identification Method was used to assess participants’ TBI history [91]. This 8-item semistructured interview assesses self-report of head or high-impact injuries to determine participants’ TBI history. Follow-up probes were used on ≤3 of the most severe injuries. Participants were excluded if they self-reported losing consciousness for 30 minutes to 24 hours (moderate TBI) or ≥24 hours (severe TBI) [92].

#### Patient-Reported Outcome Measures

All patient-reported outcome measures were collected using Qualtrics, a secure cloud-based data collection platform [90]. The Pain Outcomes Questionnaire–For Veterans (POQ-VA) [93]; Defense and Veterans Pain Rating Scale (DVPRS) [94]; and 3 single-item scales for pain, stress, and tension scale (PST) [9] were used as primary outcomes to assess the biopsychosocial impact of chronic pain. The POQ-VA and DVPRS, established VA pain measures, were administered at baseline and at the 4-, 8-, and 16-week time points. The single-item PST measures were administered at baseline and at the week 1 to week 8 time points, and again at week 16. The single-item measures for the PST were used to assess weekly fluctuations and assess short-term trends.

Veterans’ PTSD symptom severity was examined using the PTSD Checklist for the Diagnostic and Statistical Manual of Mental Disorders, Fifth Edition [95]. Secondary psychological health outcomes included the Beck Depression Inventory–II (BDI-II), which was used to measure veterans’ depression severity [96]. Veteran sleep disturbance was measured using the Pittsburgh Sleep Quality Index (PSQI) [97]. The Perceived Stress Scale (PSS) measured veterans’ self-reported stress levels [98]. Finally, health-related QOL (HRQoL) was measured using the 12-Item Short Form Health Survey (SF-12) [99]. Descriptions, number of items, response scales, and scoring are described elsewhere [9,88]. The PTSD Checklist for the Diagnostic and Statistical Manual of Mental Disorders, Fifth Edition; PSQI; PSS; SF-12; and BDI-II were administered at baseline and at the 4-, 8-, and 16-week time points (Multimedia Appendix 2).

#### Relationship Measures

Assessment of dyads incorporated the Revised Dyadic Adjustment Scale (RDAS), which is a measure of relationship satisfaction [100]. The Self-Compassion Scale (SCS) was used for assessing the compassion of participants during difficult times [101], and the Compassionate Love Scale (CLS) was used to assess compassion and altruistic love for close family and friends [102]. The RDAS, SCS, and CLS were administered at baseline and at the 4-, 8-, and 16-week time points.

#### Qualitative Measures

Qualitative semistructured interviews were conducted to elicit rich descriptions of participant experiences accessing and using MR, including their overall impressions, perceived outcomes, and recommendations for program enhancement. Given the body-mind focus of the MR program, the study applied the biopsychosocial model as a guiding framework to explore the experiences of the program participants, recognizing the multidimensionality of health and the interrelationships among the biological, psychological, and social components that contribute to one’s health and well-being [103,104].

### Onboarding Procedures

Block stratified randomization (site, concurrent PTSD, and chronic pain treatment) was used to assign consented dyads to the MR or waitlist control arms. Participants were then emailed a link to the MR website to create a profile and complete onboarding procedures. Participants who stagnated in the onboarding process were contacted up to 3 times, and study staff were available to assist participant dyads to complete onboarding. Following successful completion of the MR onboarding process, participant dyads were emailed a Qualtrics link to complete baseline survey and demographic measures. Dyads that completed the baseline survey were then unblinded to their assigned study condition.

### Qualitative Interview Procedure

Participant interviews were scheduled after the first 2 months after completion of the study intervention. Participant dyads completed a structured interview in person or over the phone to determine their eligibility and complete the informed consent process. Interviews were scheduled for 60 minutes, and consisted of 6 semistructured questions to allow for probing of the main topics. The main topics encompassed participants’ experiences participating in the MR program and using its various tools (eg, which tools were used and how), and suggestions on improving the use of MR and making it available within the VA. Data collection for the qualitative analysis began in January 2020 and was completed in May 2022.

### Data Analyses

#### Descriptive Analyses

Descriptive data are presented as frequencies and percentages for categorical data, means and SDs for normally distributed continuous data, or medians and IQRs for nonnormally distributed continuous data. Baseline group comparisons were performed using 2-tailed chi-square tests, independent-samples *t* tests or Mann-Whitney *U* tests, and Fisher exact tests. Descriptive analyses and group comparisons were conducted using SAS (version 9.4; SAS Institute) [105].

#### Missing Data Analysis

Missing item-level scale data were imputed using predictive mean matching, a method that selects closest “donor” observations based on characteristics of sex, age, race, educational attainment, relationship status, relationship duration, computer and internet use, treatment group, and survey completion time point. Missing data were not imputed if >50% of the items were missing from a scale. Missing data imputation was additionally performed for impossible observations (eg, negative time asleep) for the PSQI instrument. Predictive mean matching was performed using the *MICE* package in R (version 4.1.2; R Foundation for Statistical Computing) [105-107].

#### Mixed Models for Primary and Secondary Outcomes

Linear mixed models were constructed for primary (pain) and secondary outcomes with fixed-effects terms for treatment group (MR vs waitlist control) and time (as days since activation in the protocol) and a treatment group × time interaction term to test whether the rate of change in the instrument score differed by treatment group. Random effects for the intercept and time were also included in all models. Mixed model analyses were conducted using SAS (version 9.4) [105]. The α *P* value for statistical significance was set at .05 for the primary outcome (pain) and its subscales and .01 for the secondary outcomes. Borderline statistical significance was considered to be <.10. Standardized mean difference effect size recommendations were adopted from a preregistered systematic review and meta-analysis [108] of pain management interventions to improve affect that align with MR and mindfulness principles (≤0.32=small; 0.33-0.55=moderate; ≥0.56=large).

#### Qualitative Analysis

Data content analysis followed inductive and deductive coding methods. A qualitative codebook consisting of known constructs from the literature and those that emerged inductively from the data was created. Emerging codes were added until no new codes emerged from the data. In total, 2 trained qualitative researchers coded the data using the qualitative data management software ATLAS.ti (version 22; ATLAS.ti Scientific Software Development GmbH) [109]. To establish intercoder reliability at 80% [110,111], 2 qualitative researchers coded 20% of the interviews (initially every fifth transcript and then randomly to ensure coding consistency) separately and then compared codes to determine percentage of agreement [112]. Coded text in ATLAS.ti (version 22) was exported and further analyzed in Microsoft Excel 365 spreadsheets (Microsoft Corp) to develop themes. To enhance credibility, interim findings were presented routinely during quarterly stakeholder meetings to garner stakeholder input and facilitate refinement of findings.

## Results

### Participant Characteristics

**Figure 1:**
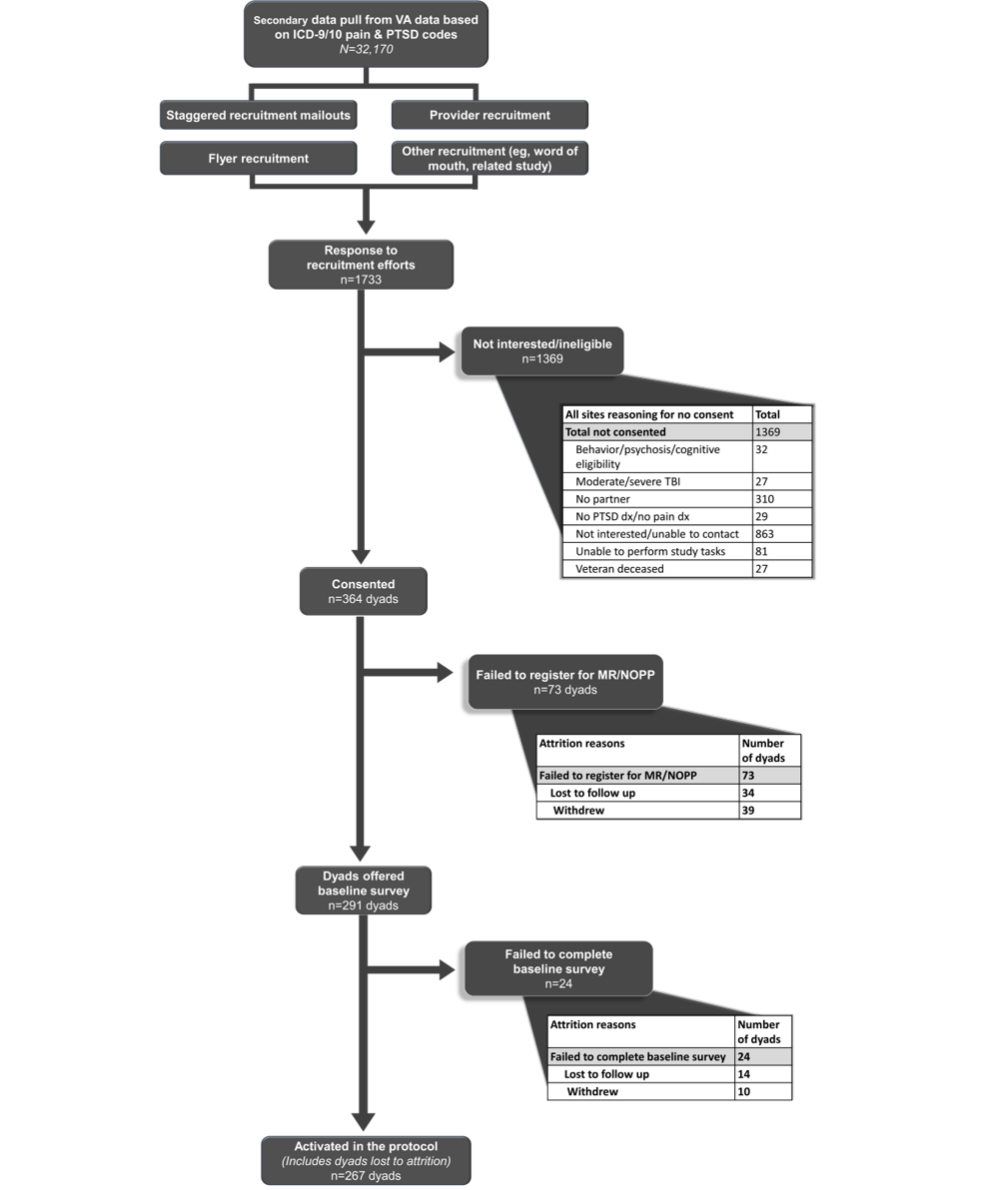
Mission Reconnect recruitment flow diagram. ICD: International Statistical Classification of Diseases and Related Health Problems; MR: Mission Reconnect; NOPP: notice of privacy practices; PTSD: posttraumatic stress disorder; TBI: traumatic brain injury; VA: Department of Veterans Affairs.

A flow diagram of the recruitment process leading to baseline survey completion has been published elsewhere (and can be reproduced) under the terms of Creative Commons Attribution 4.0 license [9,88] (Figure 1). Demographic information was examined for veterans in the MR (n=140) and waitlist control (n=136) conditions who completed baseline surveys. Veterans (n=276) had an average age of 56.54 (SD 13.74) years; were primarily male (201/276, 72.8%), White (198/276, 71.7%), non-Hispanic (236/276, 85.5%), and married or partnered (207/276, 75%); had attended or completed college or vocational school (256/276, 92.8%); and reported daily computer (167/276, 60.5%) and internet (222/276, 80.4%) use. No veteran differences were observed between the MR and waitlist control conditions across demographic characteristics (*P*≥.38 in all cases).

Partners in the MR (n=138) and waitlist control (n=134) conditions were also examined. Partners (n=272) had an average age of 52.56 (SD 14.12) years; were primarily female (222/272, 81.6%), White (206/272, 75.7%), non-Hispanic (244/272, 89.7%), and married or partnered (212/272, 77.9%); had attended or completed college or vocational school (226/272, 83.1%); and reported daily computer (177/272, 65.1%) and internet (239/272, 87.9%) use. No partner demographic differences were observed between the MR and waitlist control conditions (*P*≥.19 in all cases). Demographic characteristics by study condition are presented in Table 1 [88]. Veteran-selected partners in the sample represented four basic categories: (1) partner or spouse, (2) other family member (child or sibling), (3) friend or caregiver, or (4) not identified. A plurality of the partners were partners or spouses in the treatment (96/196, 49%) and waitlist control (76/167, 45.5%) groups. Statistics for partner categories are illustrated in Table 2. Descriptive statistics for patient-reported outcome measures by condition are reported in Table 3.

**Table 1:** Baseline demographic characteristics of participant dyads by study arm^a^.

Characteristic			Veterans							Partners							
			Mission Reconnect (n=140)		Waitlist control (n=136)		*P* value^b^			Mission Reconnect (n=138)			Waitlist control (n=134)			*P* value	
Age (y), mean (SD)			55.80 (14.13)		57.28 (13.35)		.38			52.20 (13.72)			52.92 (14.57)			.68	
**Sex, n (%)**								.48									.19
	Female	39 (27.9)		33 (24.3)					107 (77.5)			115 (85.8)					
	Male	99 (70.7)		102 (75)					29 (21)			17 (12.7)					
	Intersex	1 (0.7)		0 (0)					1 (0.7)			1 (0.7)					
	Missing or declined to respond	1 (0.7)		1 (0.7)					1 (0.7)			1 (0.7)					
**Race, n (%)**								.94									.97
	African American or Black	19 (13.6)		22 (16.2)					20 (14.5)			21 (15.7)					
	American Indian or Alaska native	3 (2.1)		1 (0.7)					1 (0.7)			0 (0)					
	Asian	1 (0.7)		1 (0.7)					5 (3.6)			4 (3)					
	Multiracial	9 (6.4)		8 (5.9)					3 (2.2)			2 (1.5)					
	Native Hawaiian or Pacific Islander	0 (0)		0 (0)					1 (0.7)			1 (0.7)					
	White	102 (72.9)		96 (70.6)					104 (75.4)			102 (76.1)					
	Other	4 (2.9)		6 (4.4)					2 (1.4)			1 (0.7)					
	Missing or declined to respond	2 (1.4)		2 (1.5)					2 (1.4)			3 (2.2)					
**Ethnicity, n (%)**								.43									.94
	Hispanic	13 (9.3)		10 (7.4)					11 (8)			9 (6.7)					
	Non-Hispanic	121 (86.4)		115 (84.6)					123 (89.1)			121 (90.3)					
	Missing or declined to respond	6 (4.3)		11 (8.1)					4 (2.9)			4 (3)					
**Marital status, n (%)**								.48									.65
	Married or partnered	109 (77.9)		98 (72.1)					111 (80.4)			101 (75.4)					
	Divorced, separated, or widowed	26 (18.6)		33 (24.3)					13 (9.4)			15 (11.2)					
	Single or never married	4 (2.9)		3 (2.2)					12 (8.7)			15 (11.2)					
	Missing or declined to respond	1 (0.7)		2 (1.5)					2 (1.4)			3 (2.2)					
**Educational level, n (%)**								.81									.30
	Lower than high school	0 (0)		0 (0)					0 (0)			1 (0.7)					
	High school	11 (7.9)		6 (4.4)					18 (13)			24 (17.9)					
	Some college or vocational school	36 (25.7)		39 (28.7)					44 (31.9)			30 (22.4)					
	Associate’s degree	35 (25)		29 (21.3)					20 (14.5)			27 (20.1)					
	Bachelor’s degree	32 (22.9)		35 (25.7)					33 (23.9)			27 (20.1)					
	Graduate degree	26 (18.6)		25 (18.4)					22 (15.9)			23 (17.2)					
	Missing or declined to respond	1 (0.7)		2 (1.5)					1 (0.7)			2 (1.5)					
Daily computer use, n (%)			87 (62.1)		80 (58.8)		.74			94 (68.1)			83 (61.9)			.51	
Daily internet use, n (%)			113 (80.7)		109 (80.1)		.83			120 (87)			119 (88.8)			.89	
**Years in relationship with partner, n (%)**								.64									.84
	<10	50 (35.7)		42 (30.9)					44 (31.9)			39 (29.1)					
	10-29	48 (34.3)		52 (38.2)					52 (37.7)			53 (39.6)					
	≥30	39 (27.9)		41 (30.1)					39 (28.3)			41 (30.6)					
	Missing or declined to respond	3 (2.1)		1 (0.7)					3 (2.2)			1 (0.7)					

^a^Percentages may not add up to 100 exactly due to rounding error.

^b^*P* values obtained from 2-tailed *t* test, chi-square test, or Fisher exact test.

**Table 2:** Partner participant relationship to the veteran (n=363).

Relationship to the veteran	Mission Reconnect, n (%)	Waitlist control, n (%)	Total, n (%)
Partner, spouse, or significant other	96 (26.4)	76 (20.9)	172 (47.4)
Other family member (eg, child or sibling)	12 (3.3)	13 (3.6)	25 (6.9)
Friend or caregiver	6 (1.7)	6 (1.7)	12 (3.3)
Unknown	82 (22.6)	72 (19.8)	154 (42.4)
Total	196 (54)	167 (46)	363 (100)^a^

^a^One dyad was not assigned to a study arm before they withdrew.

**Table 3:** Baseline veteran- and partner-reported outcome mean scores by study arm.

Scores				Cronbach α		Veterans^a^							Partners				
						Mission Reconnect		Waitlist control		*P* value^b^		Mission Reconnect			Waitlist control		*P* value
**Pain**																	
	**POQ-VA^c^**																
		Total, mean (SD)	0.87		97.25 (35.64)		87.57 (31.88)		.02^d^		N/A^e^			N/A		N/A	
		Pain intensity, mean (SD)	—^f^		6.25 (1.91)		6.07 (1.92)		.45		N/A			N/A		N/A	
		ADL^g^ interference, median (IQR)	0.93		8.00 (1.50-20.00)		5.50 (2.00-19.00)		.35		N/A			N/A		N/A	
		Kinesiophobia, mean (SD)	0.55		11.38 (4.95)		10.88 (4.88)		.41		N/A			N/A		N/A	
		Mobility interference, mean (SD)	0.89		21.63 (11.54)		19.78 (10.78)		.17		N/A			N/A		N/A	
		Negative affect, mean (SD)	0.86		31.93 (11.59)		28.61 (11.50)		.02^e^		N/A			N/A		N/A	
		Vitality, mean (SD)	0.76		20.60 (5.74)		18.21 (5.71)		<.001^e^		N/A			N/A		N/A	
	**DVPRS^h^, mean (SD)**																
		Pain intensity	—		5.91 (1.69)		5.81 (1.80)		.64		N/A			N/A		N/A	
		Activity interference	—		6.15 (2.29)		6.07 (2.36)		.77		N/A			N/A		N/A	
		Sleep interference	—		6.25 (2.73)		5.65 (2.81)		.07		N/A			N/A		N/A	
		Mood	—		6.36 (2.58)		5.91 (2.54)		.14		N/A			N/A		N/A	
		Stress	—		6.29 (2.71)		5.96 (2.54)		.29		N/A			N/A		N/A	
	**PST^i^, median (IQR)**																
		Pain intensity	—		3.00 (1.00)		3.00 (1.00)		.19		N/A			N/A		N/A	
		Muscle tension	—		4.00 (1.00)		4.00 (2.00)		.50		N/A			N/A		N/A	
		Stress	—		3.00 (1.00)		3.00 (2.00)		.06		N/A			N/A		N/A	
**Psychological, mean (SD)**																	
	PTSD^j^ (PCL-5^k^)		0.94		45.06 (15.92)		42.25 (16.57)		.15		N/A			N/A		N/A	
	Depression (BDI-II^l^)		0.93		27.05 (13.10)		25.13 (12.58)		.22		N/A			N/A		N/A	
	Stress (PSS^m^)		0.88		23.67 (6.77)		22.49 (6.65)		.14		N/A			N/A		N/A	
	Sleep (PSQI^n^)		0.80		12.84 (2.92)		12.79 (3.12)		.90		N/A			N/A		N/A	
**Quality of life, mean (SD)**																	
	Physical health (SF-12^o^)		0.84		31.30 (8.11)		31.55 (7.38)		.63		N/A			N/A		N/A	
	Mental health (SF-12)		0.82		34.44 (8.66)		36.05 (8.74)		.12		N/A			N/A		N/A	
**Relationship**																	
	Satisfaction (RDAS^p^): total, mean (SD)		0.86		3.35 (0.72)		3.44 (0.66)		.29		3.42 (0.66)			3.52 (0.67)		.22	
	Cohesion, mean (SD)		0.77		2.73 (0.96)		2.94 (1.05)		.10		2.86 (0.87)			2.86 (0.86)		.08	
	Consensus, median (IQR)		0.80		3.67 (3.0-4.17)		3.83 (3.33-4.17)		.47		3.83 (1.00)			4.00 (0.73)		.01^a^	
	Satisfaction, median (IQR)		0.78		3.75 (3.25-4.25)		3.75 (3.25-4.25)		.61		3.75 (1.00)			3.75 (1.00)		.71	
	Self-compassion (SCS^q^): total, mean (SD)		0.94		2.60 (0.77)		2.71 (0.80)		.22		3.21 (0.78)			3.24 (0.76)		.71	
	Common humanity, mean (SD)		0.79		2.71 (0.93)		2.83 (0.95)		.31		3.29 (1.01)			3.39 (1.00)		.39	
	Isolation, mean (SD)		0.82		2.56 (1.05)		2.61 (1.11)		.70		3.24 (1.08)			3.16 (0.97)		.55	
	Mindfulness, mean (SD)		0.80		2.88 (0.88)		3.09 (0.95)		.05		3.46 (0.95)			3.52 (0.88)		.65	
	Overidentification, mean (SD)		0.79		2.65 (1.04)		2.75 (0.95)		.44		3.19 (1.00)			3.20 (0.94)		.92	
	Self-kindness, mean (SD)		0.84		2.44 (0.91)		2.63 (1.00)		.09		3.11 (0.93)			3.18 (0.97)		.55	
	Self-judgment, mean (SD)		0.84		2.43 (0.97)		2.44 (0.96)		.83		3.02 (0.99)			3.06 (0.93)		.93	
	Compassion for others (CLS^r^), median (IQR)		0.95		5.81 (4.90-6.55)		5.95 (4.77-6.64)		.22		6.29 (0.97)			6.19 (1.09)		.16	

^a^3 veterans were excluded for incomplete data.

^b^*P* values were obtained from independent-sample 2-tailed *t* tests or Mann-Whitney *U* tests.

^c^POQ-VA: Pain Outcomes Questionnaire–For Veterans.

^d^*P*<.05.

^e^N/A: not applicable.

^f^Cronbach α cannot be calculated for single-item measures.

^g^ADL: activity of daily living.

^h^DVPRS: Defense and Veterans Pain Rating Scale.

^i^PST: pain, stress, and tension scale.

^j^PTSD: posttraumatic stress disorder.

^k^PCL-5: PTSD Checklist for the Diagnostic and Statistical Manual of Mental Disorders, Fifth Edition.

^l^BDI-II: Beck Depression Inventory–II.

^m^PSS: Perceived Stress Scale.

^n^PSQI: Pittsburgh Sleep Quality Index.

^o^SF-12: 12-Item Short Form Health Survey.

^p^RDAS: Revised Dyadic Adjustment Scale.

^q^SCS: Self-Compassion Scale.

^r^CLS: Compassionate Love Scale.

### Primary Outcomes

**Figure 2:**
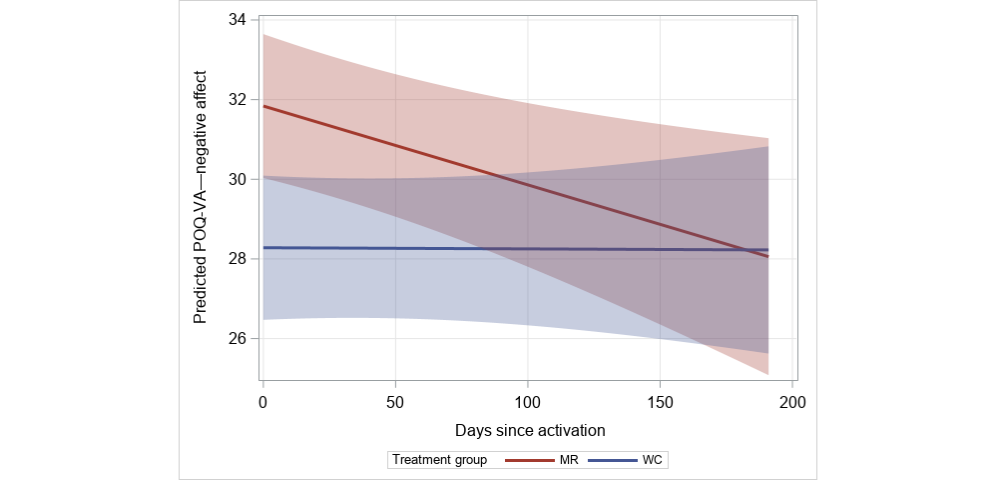
Predicted values for Pain Outcomes Questionnaire–For Veterans (POQ-VA) negative affect subdomain scores over time by treatment group. MR: Mission Reconnect; WC: waitlist control.

**Figure 3:**
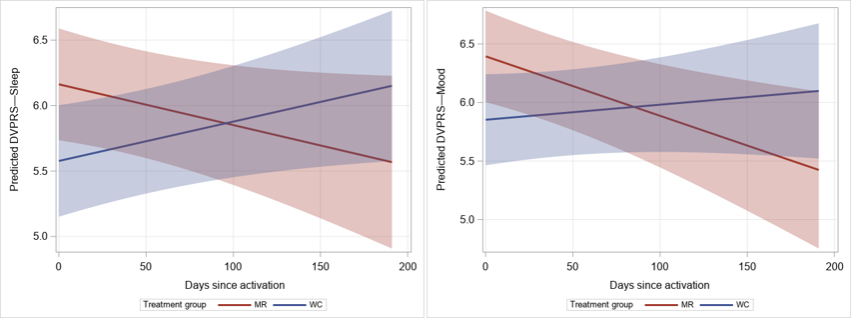
Predicted values for Defense and Veterans Pain Rating Scale (DVPRS) sleep (left panel) and mood (right panel) subdomain scores over time by treatment group. MR: Mission Reconnect; WC: waitlist control.

Improvements in total pain were not observed as measured using the POQ-VA (*P*=.38), DVPRS (*P*=.17), or PST (*P*=.19). In analyses of the subdomains of the POQ-VA (pain rating, impairment of mobility, *P*=.53; impairment of activities of daily living, *P*=.69; impairment of vitality, *P*=.17; and fear, *P*=.13), no significant improvements were detected over time, although a significant decline in the MR group relative to the waitlist control group was observed for negative affect (β=–.020; SE 0.010; *P*=.049; Table 4 and Figure 2). Similarly, while there was no change in pain interference with activity or stress for either group as measured using the DVPRS, there were significant reductions in pain interference in sleep (β=–.006; SE 0.002; *P*=.008) and mood (β=–.006; SE 0.002; *P*=.008) among the MR group compared to the waitlist control group (Figures 3A and 3B, respectively). For PTSD, no treatment effect was observed for the MR group compared to the waitlist control group over the follow-up period (*P*=.48).

**Table 4:** Fixed-effects estimates for select patient-reported Mission Reconnect (MR) outcomes.

Patient-reported outcome measure				Fixed effects				SMD^a^
				β (SE; 95% CI^b^)		*P* value		
**Veterans**								
		POQ^c^ (negative affect): time × treatment group (MR vs WC^d^)	–0.020 (0.010; –0.039 to 0.000)		.049		–0.13	
		DVPRS^e^ (sleep): time × treatment group (MR vs WC)	–0.006 (0.002; –0.011 to –0.002)		.008		–0.16	
		DVPRS (mood): time × treatment group (MR vs WC)	–0.006 (0.002; –0.011 to –0.002)		.008		–0.16	
		PST^f^ (stress): time × treatment group (MR vs WC)	–0.002 (0.001; –0.004 to 0.000)		.10		–0.10	
		SF-12^g^ (mental health): time × treatment group (MR vs WC)	0.017 (0.008; 0.002 to 0.033)		.03		0.13	
		SCS^h^ (overidentification): time × treatment group (MR vs WC)	0.002 (0.001; 0.000 to 0.004)		.04		0.14	
**Partners**								
	RDAS^i^ (total score): time × treatment group (MR vs WC)			0.021 (0.008; 0.005 to 0.038)		.01		0.19
	RDAS (total consensus): time × treatment group (MR vs WC)			0.010 (0.004; 0.002 to 0.019)		.02		0.16
	RDAS (consensus [affection subdomain]): time × treatment group (MR vs WC)			0.006 (0.002; 0.002 to 0.010)		.007		0.20
	RDAS (total satisfaction): time × treatment group (MR vs WC)			0.004 (0.002; 0.000 to 0.009)		.07		0.07
	RDAS (satisfaction [conflict subdomain]): time × treatment group (MR vs WC)			0.005 (0.001; 0.002 to 0.007)		.001		0.22

^a^SMD: standardized mean difference.

^b^Fixed-effects estimates for interaction term of time (per day) × treatment group (MR vs waitlist control) indicating that the rate of change over time differed between the MR group and the waitlist control group. Only outcomes that were found to be statistically or borderline significant (*P*≤.10) are included in the table.

^c^POQ: Pain Outcomes Questionnaire–For Veterans.

^d^WC: waitlist control.

^e^DVPRS: Defense and Veterans Pain Rating Scale.

^f^PST: pain, stress, and tension scale.

^g^SF-12: 12-Item Short Form Health Survey.

^h^SCS: Self-Compassion Scale.

^i^RDAS: Revised Dyadic Adjustment Scale.

### Secondary Outcomes

For total stress measured using the PST, we observed a reduction in stress in the MR group relative to the waitlist control group that was of borderline significance (β=–.002; SE 0.001; *P*>.99; Table 4); however, no effect of the MR intervention was observed for stress as measured using the PSS (*P*=.18). We also did not observe an effect of the MR intervention on other secondary outcomes, including muscle tension (PST; *P*=.73), depression (BDI-II; *P*=.20), sleep (PSQI; *P*=.24), and physical health (SF-12; *P*=.84). Mental health, as assessed using the SF-12, improved in the MR group relative to the waitlist control group (β=.017; SE 0.008; *P*=.03; Table 4), although this was considered to be of borderline significance (with the adjusted *P* value).

Among veterans, the average total SCS score did not differ by treatment group (*P*=.13). Similar results were found for the partners, with no difference by treatment group (*P*=.70). Many of the subdomain scores [self-kindness (*P*=.37), self-judgment (*P*=.26), common humanity (*P*=.33), isolation (*P*=.17), and mindfulness (*P*=.11)] did not differ by treatment group for either veterans or partners. For veterans, overidentification with personal failures and shortcomings significantly improved in the MR group compared to the waitlist control grou*p* (β=.002; SE 0.001; *P*=.04; Table 4). No effect of the MR intervention was observed for overidentification among partners (*P*=.76). Similarly, no effect was observed for either veterans or partners (*P*=.48) on the CLS (*P*=.24) and for veterans on the RDAS (*P*=.72).

**Figure 4:**
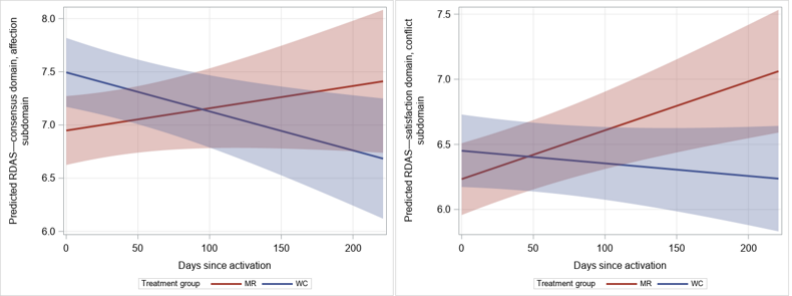
Predicted values for Revised Dyadic Adjustment Scale (RDAS) affection (left panel) and conflict (right panel) subdomain scores among partners over time by treatment group. MR: Mission Reconnect; WC: waitlist control.

We observed significant or borderline significant findings on the RDAS (ie, relationship satisfaction) for partners. Overall relationship satisfaction increased for partners in the MR group relative to those in the waitlist control group (β=.02; SE 0.01; *P*=.01; Table 4), as well as in the domains of consensus (β=.01; SE 0.004; *P*=.02) and satisfaction (β=.004; SE 0.002; *P*=.07), although these results were of borderline significance. These findings are likely driven by significant improvements in the affection (β=.01; SE 0.002; *P*=.007) and conflict (β=.005; SE 0.001; *P*=.001) subdomains of the consensus and satisfaction domains (Figure 4). No effect of the treatment was observed on the subdomains of consensus (decision-making, *P*=.14 and values, *P*=.63) and satisfaction (stability, *P*=.72), as well as the cohesion domain and its subdomains (activities, *P*=.11 and discussion, *P*=.16).

### MR Satisfaction

Veteran and partner satisfaction with MR was relatively high at weeks 4, 8, and 16. For all 11 satisfaction items, median ratings ranged from 7 to 9 on a 10-point scale, with higher scores indicating greater satisfaction. Participant experiences with MR were further examined using qualitative data, as presented in the following section.

### Qualitative Results

The interview subsample reflected the larger randomized controlled trial sample, representing veterans (n=35) with an average age of 53.94 (SD 12.76) years who were primarily male (28/35, 80%), non-Hispanic (29/35, 83%), and married or partnered (25/35, 71%); had attended or completed college or vocational school (32/35, 91%); and reported daily computer (25/35, 71%) and internet (29/35, 83%) use. Partner interviewees (n=35) had an average age of 49.89 (SD 13.88) years; were primarily female (29/35, 83%), non-Hispanic (31/35, 89%), and married or partnered (26/35, 74%); had attended or completed college or vocational school (33/35, 94%); and reported daily computer (21/35, 60%) and internet (29/35, 83%) use (Multimedia Appendix 3).

#### Overall Impressions of MR

The overall impression from participant qualitative interviews was that veterans and their partners valued the program, which corresponded with the quantitative satisfaction data. They reported that the program was inclusive, supported skill development, promoted a sense of positivity, and reinforced users’ existing practices. Most participants reported scheduling MR activities and incorporating them into their daily routines. Nearly half reported using the app. Usability and navigation issues were relevant to user experience, consistent with similar apps. There were reports that indicated that the benefits such as portability and remote access from anywhere were highly valued. In addition, navigation and accessibility were relevant; however, reports were mixed. Participants requested guidance and support, which was provided as needed. An in-person component was recommended for programmatic implementation.

Barriers to using MR included personal characteristics or situations or not finding time to integrate the techniques into their lives. In addition, as with most video and audio-based interventions, length of time to complete the content was an issue for users. Other recommendations included providing additional content, such as shorter videos, content for family (including kids), additional meditations, and advanced material; accommodating different learning styles; and organizing content by symptoms and having a questionnaire to help users identify what they should prioritize. Facilitators of using MR included having a conducive environment, experiencing benefits, and the program content and structure.

#### Effect of MR

Veterans and their partners reported that the program was helpful and yielded positive outcomes. Veterans specifically indicated alleviation of symptoms related to pain, anxiety, and PTSD. In addition, veterans and partners reported improvement in QOL and influence on their relationship and communication. Multimedia Appendix 4 quantitatively counts veterans’ and their partners’ predominantly positive endorsement of outcome domains, whereas Multimedia Appendix 5 illustrates exemplary quotes by subdomain and participant type across all dimensions of the biopsychosocial model, including biological effects (ie, pain and sleep), psychological effects (ie, PTSD symptoms and anxiety), and social effects (ie, relationships with others). These domains and subdomains are illustrated in the following paragraphs.

#### Biological Effects: Pain and Sleep

Veterans talked about MR and their pain throughout the interviews. Some veteran participants reported that they learned activities to alleviate pain, although often the relief was temporary or inconsistent. Some participants reported trying several of the activities and obtaining skills that helped them cope with the pain. Overall, specific MR activities, such as partner massage and the activities “loosening your body” and “movement to stillness,” were mentioned as a source of muscle release and relaxation. Reduced pain was linked to an overall improved attitude and QOL. For some participants who were not able to take or preferred not taking pain medication, the CIH approaches served as an effective nonpharmacological solution.

While some indicated a reduced pain intensity, activities made pain worse for other participants. For instance, the Waking up the Body exercise that incorporates rhythmical patting of meridians was too painful for some participants to incorporate into their routine. Massage of some body parts or with too hard a touch was also reported by some to make their pain worse.

The effect of MR on sleep was discussed minimally. In general, participants felt that the MR activities helped them fall asleep more easily; a few discussed “improved sleep.” MR activities, such as intentional yawning to release tension (Reset and Refresh), guided meditation (Deep Relaxation), and swaying movement to align body and mind (Movement to Stillness) contributed to improved sleep.

#### Psychological Effects: PTSD, Anxiety, and Depression

Few participants made a direct connection between MR activities and PTSD symptoms specifically. Of those who did, participants described experiencing PTSD symptoms as being “stuck” in the past, reliving the feelings around a traumatic event. They reported a benefit to getting back in touch with and learning to live in the present. They reported being more aware and, thus, able to bring their focus to their current situation and environment. This shift in focus was reported to help limit negative PTSD symptoms. Of the participants who referenced PTSD-related nightmares, most reported using MR activities (mindfulness, quieting the mind, and massage therapy) to relieve nightmares and associated feelings.

Participants reported that MR was helpful in managing mental health symptoms, such as improving their anxiety. Veterans described using the videos and MR activities in a variety of ways (website, app, or saved to the cloud) to help manage anxiety and depression. Others reported accessing MR videos to manage PTSD symptoms during an event. Participants reported that the meditation and mindfulness activities were also helpful and created a sense of quiet and calm, although these benefits may not necessarily last beyond the practice time. Participants reported better self-awareness that ultimately helped them understand how their PTSD affects their communication. With this self-awareness, they were able to change some of their communication and, therefore, improved their relationships and a sense of connection with others. Notably, some adverse effects were attributed to the study activities. Several veterans reported that completing the study survey items, especially those measuring PTSD symptoms, created stress and, at times, exacerbated their symptoms.

#### Social Effects: Relationships With Others

Participants reported that their use of MR and the CIH activities was beneficial for the relationship with their study partner. Participants overwhelmingly talked about improved communication, reporting that their communication occurred more often and was more productive. Improved communication, connection, and understanding of each other were often cited as outcomes of their study coparticipation. MR activities required scheduling time together in otherwise busy lives. This scheduled time was seen as beneficial to reconnecting with partners. Activities often required doing things outside their daily patterns, creating more interaction between dyads and promoting more touch with partners. The interaction among partners indicated an improved connection and understanding. Participants reported that MR activities brought about a better understanding of the self (eg, meditation). This enhanced awareness of their own feelings facilitated improved communication and understanding among partners.

#### Effects on QOL

Several participants made general statements about MR’s impact on their general QOL, such as “My life is better now.” Participants spoke to the general improved QOL experience through using the MR platform and CIH activities. Those who spoke of improved QOL often spoke of adopting a positive attitude, especially at the beginning of the day; reconnecting or “centering” oneself; and feeling more relaxed.

## Discussion

### Principal Findings

Making nonpharmacological treatments such as CIH available remotely can help reduce overreliance on pain medications and promote patient-centered care, and can increase access and use of nonpharmacological pain treatments [113]. There is evidence that CIH can be used for pain management with little evidence of negative effects and can be easily implemented remotely with impactful results [21]. The purpose of this waitlist control mixed methods randomized controlled trial was to describe the physical, psychological, and social outcomes associated with the use of a self-directed remotely delivered CIH education intervention for veterans with comorbid chronic pain and PTSD and their partners.

Although participants reported high levels of satisfaction with MR, comparisons did not show an effect on our primary outcome, pain, as measured using the total POQ-VA score, DVPRS, or PST. Our findings diverge from the literature establishing the use of CIH, including massage therapy, movement, and mindfulness, to improve pain outcomes such as pain intensity and physical functioning in veteran and nonveteran populations [12,13,15,19]. We found significant small–effect size improvements on the subdomains of negative affect (POQ-VA) and pain interference on mood and sleep (DVPRS). We also observed a small–effect size improvement for mental HRQoL (SF-12 score) consistent with a meta-analysis that found a comparable small effect size improvement for massage therapy and improved overall HRQoL (using the SF-12 and 36-Item Short Form Health Survey) [24]. However, low certainty of evidence has been found for massage therapy in improving overall HRQoL among people with chronic pain [38]. For partners, the treatment effect was nearly statistically significant for overall relationship satisfaction and the domains of consensus and satisfaction (RDAS), with small effect sizes being observed. Statistically significant small–effect size improvements in the subdomains of affection and conflict were noted.

In contrast to our results, in a review of 2 randomized trials for co-occurring chronic pain and PTSD, pooled analyses found nonsignificant changes in pain intensity and interference but significant improvement in PTSD symptoms [72]. Interestingly, the authors noted that interventions targeting co-occurring chronic pain and PTSD typically target the latter, which may explain the limited effects on pain-related outcomes. The nonsignificant improvement in pain reduction during MR was inconsistent, with specific meta-analytic evidence supporting massage therapy over passive and active interventions for chronic pain overall [24,114]. However, the broader literature on massage therapy for pain management is mixed and inconclusive. Certain meta-analyses examining specific chronic pain conditions have found moderate evidence for low back [12,13] and neck pain [32,37] and strong evidence for shoulder pain [31]. An additional meta-analysis examining massage therapy in pooled analyses with other types of CIH found support for pain reduction [17]. Conversely, a recent Cochrane review [38] and evidence maps [15,35] indicate less certainty regarding massage therapy for pain reduction. Giannitrapani et al [15] found support for the benefits of massage therapy on certain types of pain (eg, general chronic pain) but unclear evidence for others (eg, neck pain), with variability across studies, pain location, and massage type. Although evidence in the literature is compelling, our powered randomized controlled trial did not find strong support for partnered massage for pain reduction. Our findings could be resultant of not focusing on a location-specific pain condition or the partnered delivery of massage versus professional massage, as is often practiced in CIH trials.

Mindfulness-based interventions are often used with chronic pain populations given their low intensity and limited adverse outcomes [12,114]. This study did not support existing meta-analytic evidence for pain reduction among people with chronic neck [46] and low back pain, [48,56-61] or depression reduction [65] among general chronic pain despite mindfulness being an important MR component. However, the MR intervention was associated with significant small–effect size improvements in pain interference with mood and sleep. Although consistent, previous meta-analyses have found medium–effect size improvements in pain interference for massage therapy compared to inactive controls [62,63]. Previous reviews support massage therapy and mindfulness-based stress reduction techniques as moderately effective at reducing pain and improving function over the short term [22,24-26] but may not produce similar moderate to large effects for other types of pain [22,36]. Furthermore, this evidence is largely based on immediate, short-term, and pretest-posttest intervention effects, with limited evidence from studies to support follow-up effects [24,31,32,37,61]. As mentioned previously, these distinctions may inform why we did not find a significant reduction in pain as our population did not present a homogenous pain source. There is limited published research on the effects of nonpharmacologic therapies on subdomains of pain (negative affect and pain interference on mood and sleep), so these results warrant further examination.

Significant improvements for PTSD were not observed in this trial. This is inconsistent with systematic review and meta-analytic literature that indicates the promise of CIH approaches, including mindfulness and mind-body interventions, for PTSD and mental health symptoms (eg, depression and anxiety), which are directly relevant for MR [67,70,73,74,76]. However, several of these studies suggest caution due to possible publication bias [69] as well as studies being underpowered [74,79,80] and having low methodological quality [66,68,70,73]. Furthermore, studies have also demonstrated that evidence from randomized controlled trials examining mindfulness and mind-body interventions for PTSD symptom improvement is generally positive [48,67] or mixed [66,69,80]. Perhaps part of the reason for inconsistent findings across trials that include MR is that CIH interventions may require tailoring for these unique patient populations [68].

Our findings do not support the literature suggesting that CIH, such as meditation and breathing exercises, can be an effective self-management approach to PTSD symptoms for veterans [79,80]. However, Haider et al [79] found consistent evidence in the literature suggesting improvement in PTSD symptoms for veterans. Cushing and Braun [80] found evidence for improving PTSD and mental health symptoms, including depression, as well as evidence for improved sleep quality, which was supported in this trial. It is notable that sample sizes, methodological rigor, and limited reporting of effect sizes are identified as limiting factors in efforts to compare results across trials [79,80].

It is critical to note that the findings of this trial are relatively inconsistent with those of previous MR studies. A pilot study of postdeployment National Guard service members found significant pretest-posttest improvements in pain, tension, and depression. A follow-up randomized controlled trial found significant improvement in veterans’ pain, PTSD, depression, sleep, tension, and self-compassion from baseline to after MR. The findings of our study only replicated improvements in sleep and a nonsignificant trend for negative affect (eg, depression). Effect sizes were unavailable for direct comparison. Of note, the differences among these studies may be accounted for by the fact that previous MR trials used nonclinical samples, which may have less severe conditions than the co-occurring pain and PTSD criteria for our trial. Still, our study did expand the MR evidence base supporting positive relationship outcomes, including small–effect size improvements in affection and reduced conflict, as well as a borderline significant effect for relationship satisfaction.

Convergences and divergences of quantitative and qualitative data were documented in the findings. In interviews, veterans and partners indicated benefits of the program and reported ease of incorporation of activities into their daily life activities. Veterans mainly highlighted physical benefits of the program specifically related to pain and PTSD symptoms. Veterans and partners indicated positive effects of the program on mental health, social health, and QOL. In addition, they mentioned that they slept better and felt more energized and refreshed. These findings are in line with those of previous publications on the benefits of remotely delivered CIH for veterans. Consistent with the quantitative data, the survey and interview data indicated improvement in pain interference and overidentification for veteran participants, as exemplified by the following quote:

Yeah, I think it’s helped me—you know, being able to connect with my wife. It’s helped me to feel more comfortable sharing with her when I have thoughts or struggles, or I’m just kind of feeling out of it. You know, I feel like I can—because we’re more connected, I can share with her.

Interestingly, interview data indicated improvement for partners in these same domains, although the quantitative data did not indicate differences among partners between the study arms. In addition, interview data indicated meaningful improvement for overall veteran-reported pain and PTSD symptoms, which was not noted in the quantitative findings. The inconsistencies between the quantitative and qualitative findings highlight a need for future research to identify appropriate CIH measures for veteran and partner populations [115,116].

The VHA is well positioned to provide nonpharmacological treatment for pain [116,117]. Nonpharmacological treatment for pain is associated with reducing opioid use and overall improved outcomes, although the quality [14,18,118] of evidence varies. Veterans who use VHA services have a greater likelihood of receiving nonpharmacological treatment, including educational classes and mind-body therapy, for chronic pain [119]. Given limitations in staffing and potential wait times for new patient appointments, MR may improve access and provide timely, patient-centered care as a fully remote intervention.

Implementation of MR as a fully remote mobile and web-based intervention has not been attempted in previous studies. It is notable that, in the study by Kahn et al [87], there was an in-person component in one of the treatment arms. We did not replicate the in-person component because the data from the aforementioned study did not indicate a significant benefit. In addition, due to the coincidence of data collection for this study with the COVID-19 outbreak, an in-person component would not have been feasible. It is possible that the lack of an in-person component impacted intervention engagement and modality use and, thereby, intervention outcomes [120].

Interview data highlighted MR’s effects on PTSD symptoms and perceived improvements in communication, relationship quality, and sense of connectedness. Participation in the program did impact pain and interference, but mainly, the program supported acquisition of coping mechanisms. With regard to effectiveness on outcomes, effects may have been related to dose response. Despite reporting an overall positive experience and satisfaction with MR, participant recommendations for improvements in usability and engagement could enhance program use and attract more sustained users.

### Future Research and Implementation

After nearly a decade, the VA continues its efforts to understand the impact of the whole health and CIH modalities on the health and well-being of veterans [116,117]. More evidence is needed to determine how to optimize the integration of whole health and CIH into health care to meet veterans’ needs and values, particularly of veterans with PTSD [121]. Furthermore, there is evidence suggesting that, when service members engage in CIH approaches for pain while on active duty, they have a lower risk of substance abuse, overdose, and suicide attempt later in life [118]. As such, promoting the use of whole health and CIH throughout the military and veteran health care trajectories may be critical for improving outcomes, particularly for veterans with pain and PTSD.

Adjunctive modalities such as MR can help veterans manage chronic pain and PTSD symptoms, but further research using pragmatic trials [122] and implementation efforts [123] are warranted. The findings warrant a tailored approach for vulnerable at-risk populations, such as veterans with comorbid chronic pain and PTSD. As we advance the science to determine the risks and benefits of CIH-based interventions specifically for PTSD, some features of CIH modalities, such as massage therapy and touch, may cause emotional discomfort for this vulnerable population. Future studies should aim to (1) increase sampling to allow for analysis of stratified pain or PTSD profiles; (2) explore divergent findings specifically related to pain, PTSD, and relationship outcomes; (3) reassess the fit of outcome measures to address sensitivity to change of outcomes, such as connectedness; (4) assess professional versus partnered massage with attention to those who experience PTSD symptoms; (5) conduct an intervention component and dosage evaluation; and (6) evaluate the CIH effects on opioid use [49] and misuse. Consideration of a standardized approach to measuring patient self-reported use and outcomes is also warranted to support advancing the science through the continued application of cross-study meta-analyses [115].

When planning future research with this vulnerable population, the increased risk of frustration with onboarding and data collection processes should be considered. Authors recommend simplifying onboarding processes and providing personal support throughout the study and increasing automation, reminders, and project navigators (electronic and human). With studies relevant to PTSD, revisiting trauma is a risk—as such, we recommend a rigorous screening and consent process that clearly prepares patients for the types of emotional, mental, and physical health data they will be providing to manage expectations and burden.

The findings indicate that an in-person component in tandem with the intervention is warranted to provide personalized support. Recommended strategies included personalized recommendations based on user preferences, needs, and progress; tailoring content to individual pain and PTSD profiles; and fostering a sense of community among users by integrating social features to create a supportive network. Future implementation will benefit from efforts to improve 508 compliance standards set for federal agencies to ensure technology features are accessible [124] for optimized usability and navigation.

### Strengths and Limitations

Although the findings are compelling, the balance of strengths and study limitations should be considered. First, this trial was launched during the COVID-19 pandemic. Consistent with the literature [120], many adjustments were required for nonpharmacological pain management trials during the pandemic; however, the remote nature of MR minimized disruption to the trial process and outcomes. Nonetheless, we acknowledge that the pandemic could have presented confounding factors (eg, social isolation and pandemic-related competing demands) that may have influenced the study findings. Second, regarding mobile and web-based delivery, the data represented 3 distinct sites; however, participants from each site represent a larger geographic area that comprises rural and nonrural areas across most of the United States. Third, participants reported that “too many” surveys were required at each time point. At the time of this study, no single instrument existed to capture the CIH patient experience. Including a single survey for CIH patient experience [123] may have limited survey burden, thus improving the reliability of the findings. Fourth, we were unable to examine dose effect (eg, more frequent or longer use of the MR program could result in improved outcomes) due to the lack of an effective measure of frequency and duration of use and lack of consistent participant reporting. Fifth, we did not further adjust for demographic variables, including those used for the missing data imputation, in the final models due to the relatively small sample size and general lack of significant findings. However, as randomization is expected to result in balanced groups, it is generally not necessary to further adjust for baseline characteristics in randomized clinical trials, and baseline demographics did not differ between groups. We also did not consider, in the final models, concurrent treatments for pain and PTSD that may have had synergetic or deleterious effects on the program’s impact on patient-reported outcomes. Sixth, regarding the qualitative results, while we achieved thematic saturation, the findings may not be generalizable to other CIH programs, and insights may have been overlooked from the veterans or partners who participated in the larger parent randomized controlled trial but did not participate in the interviews.

### Conclusions

MR offers a CIH-based intervention for veterans with chronic pain and PTSD and their partners, with significant impacts on pain interference and relationship quality. Additional design and content modification can enhance the program’s fit and use for this population and may generate greater uptake. Data findings can inform MR program enhancements, protocol design for subsequent pragmatic trials in diverse populations, and implementation of MR and other nonpharmacological CIH-based remotely delivered partnered programs in clinical settings. The findings warrant future efforts to explore the role of pain interference in pain management interventions, impacts on relationship outcomes, and cost-effectiveness of the program.

## Appendix

Multimedia Appendix 1 CONSORT-EHEALTH (Consolidated Standards of Reporting Trials of Electronic and Mobile Health Applications and Online Telehealth) checklist (V 1.6.x).

Multimedia Appendix 2 Patient-reported outcome measures administered.

Multimedia Appendix 3 Demographic characteristics of Mission Reconnect participants who completed qualitative interviews.

Multimedia Appendix 4 Outcome domains endorsed in interviews by unique respondent type.

Multimedia Appendix 5 Veteran and partner dyad quotes representing qualitative domains.

## Abbreviations

BDI-II: Beck Depression Inventory–II
CIH: complementary and integrative health
CLS: Compassionate Love Scale
CONSORT-EHEALTH: Consolidated Standards of Reporting Trials of Electronic and Mobile Health Applications and Online Telehealth
DVPRS: Defense and Veterans Pain Rating Scale
HRQoL: health-related quality of life
MR: Mission Reconnect
POQ-VA: Pain Outcomes Questionnaire–For Veterans
PSQI: Pittsburgh Sleep Quality Index
PSS: Perceived Stress Scale
PST: pain, stress, and tension scale
PTSD: posttraumatic stress disorder
QOL: quality of life
RDAS: Revised Dyadic Adjustment Scale
SCS: Self-Compassion Scale
SF-12: 12-item Short Form Health Survey
TBI: traumatic brain injury
VA: Department of Veterans Affairs
VHA: Veterans Health Administration

## Data Availability

The deidentified anonymized datasets included in this manuscript are available from the corresponding author upon reasonable request.
