# Supplementary material for: Outcomes of a Remotely Delivered Complementary and Integrative Health Partnered Intervention to Improve Chronic Pain and Posttraumatic Stress Disorder Symptoms: Randomized Controlled Trial
Source: J Med Internet Res. 2024 Oct 18;26:e57322. doi: 10.2196/57322 (PMC11530734; doi:10.2196/57322)
Supplement: Multimedia Appendix 2 [file jmir_v26i1e57322_app2.docx]

Patient-reported outcome measures administered.

| **Scale** | **Construct** | **Description** | **Items** | **Scale** | **Scoring** |
| --- | --- | --- | --- | --- | --- |
| **Pain** |  |  |  |  |  |
| POQ-VA | Pain | Pain Intensity, Interference (Mobility, Activities of Daily Living), Negative Affect, Vitality, and Fear of Movement | 19 | 0 – 10^*^ | Sum All Items – Higher scores indicate worse outcomes. |
| DVPRS | Pain | Pain Intensity, Interference (Activity, Sleep), and Impact of Psychological Health (Mood, Stress) | 5 | 0 – 10^*^ | Mean of All Items – Higher scores indicate worse outcomes. |
| PST | Pain | Pain, Muscle Tension, and Stress | 3 | 1 – *None*  5 – *Worst* | Individual Items – Higher scores indicate worse outcomes. |
| **Psychological** |  |  |  |  |  |
| PCL-5 | PTSD | Items assess individual PTSD symptoms such as intrusive re-experiencing of traumatic event, avoidance of triggers reminiscent of traumatic event, negative affect, and hyperarousal. | 20 | 0 – *Not at All*  4 – *Extremely* | Sum All Items – Higher scores indicate worse outcomes. |
| BDI-II | Depression | Items assess common depression symptoms such as sadness, guilt, and sleep and appetite problems. | 21 | 0 – 3^*^ | Sum All Items – Higher scores indicate worse outcomes. |
| PSQI | Sleep Disturbance | Items assess multiple sleep disturbance factors such as time slept, sleep quality, and barriers to sleep in the past month. | 19 | Various^*^ | Scoring algorithm used to weight and combine item responses into subscales which were then summed. Higher scores indicate worse outcomes. |
| PSS | Perceived Stress | Items assess multiple sleep disturbance factors such as nervousness, sleep quality, coping abilities, and self-confidence in the ability to handle stress. | 4 | 0 – *Never*  4 – *Very Often* | Sum All Items – Items 4 5, 7, and 8 reverse scored for interpretive consistency. Higher scores indicate worse outcomes.^+^ |
| **HRQoL** |  |  |  |  |  |
| SF-12 | Health-Related Quality of Life | Quality of life using physical status and mental health distress. | 12 | Various^*^ | Norm-based scoring algorithm used to weight and combine item responses into physical and mental health subscales. Higher scores indicate better outcomes. |
| **Relationships** |  |  |  |  |  |
| RDAS | Relationship Satisfaction | Relationship Cohesion, Consensus, and Overall Satisfaction within the Dyad. | 14 | Various^*^ | Sum All Items – Higher scores indicate better outcomes. |
| SCS | Self-Compassion | Compassion participants show themselves during difficult times. | 26 | 0 – *Almost Never*  5 – *Almost Always* | Mean of All Items – 13 items reverse scored for interpretive consistency. Higher scores indicate better outcomes.^+^ |
| CSL | Perceived Stress | Compassion and altruistic love for close family and friends | 21 | 1 – *Not at All True of Me*  5 – *Very True of Me* | Mean of All Items – Higher scores indicate better outcomes. |

^*^Item-level response options vary by domain.

^+^Reverse-scored for interpretive consistency.

BDI-II = Beck Depression Inventory-II; CLS = Compassionate Love of Close Others Scale; Defense and Veterans Pain Rating Scale = DVPRS; HRQoL = Health-Related Quality of Life; PCL-5 = Posttraumatic Stress Disorder (PTSD) Checklist for Diagnostic and Statistical Manual of Mental Disorders-Fifth Edition; POQ-VA = Pain Outcomes Questionnaire-Veterans Affairs; PSQI = Pittsburgh Sleep Quality Index; PSS = Perceived Stress Scale; PST = Pain, Stress , and Tension; Revised Dyadic Adjustment Scale = RDAS; SCS = Self-Compassion Scale; SF-12 = Short Form-12.
