# Supplementary material for: Outcomes of a Remotely Delivered Complementary and Integrative Health Partnered Intervention to Improve Chronic Pain and Posttraumatic Stress Disorder Symptoms: Randomized Controlled Trial
Source: J Med Internet Res. 2024 Oct 18;26:e57322. doi: 10.2196/57322 (PMC11530734; doi:10.2196/57322)
Supplement: Multimedia Appendix 3 [file jmir_v26i1e57322_app3.docx]

Demographic characteristics of Mission Reconnect participants who completed qualitative interviews (n=70).

| Characteristic | | Participants | |
| --- | --- | --- | --- |
|  | | Veteran | Partner |
|  |  | n=35 | n=35 |
| Age in years, mean (SD) | | 53.94 (12.76) | 49.89 (13.88) |
| **Gender, n (%)** | | | |
|  | Female | 7 (20.00%) | 29 (82.86%) |
|  | Male | 28 (80.00%) | 5 (14.29%) |
|  | Other | 0 (0.00%) | 1 (2.86%) |
| **Race, n (%)** | | | |
|  | White | 24 (68.57%) | 26 (74.29%) |
|  | African American or Black | 5 (14.29%) | 6 (17.14%) |
|  | Asian | 0 (0.00%) | 2 (5.71%) |
|  | Multiracial | 4 (11.43%) | 1 (2.86%) |
|  | Other | 1 (2.86%) | 0 (0.00%) |
|  | Missing/Decline to Respond | 1 (2.86%) | 0 (0.00%) |
| **Hispanic ethnicity, n (%)** | | | |
|  | Hispanic or Latino | 6 (17.14%) | 3 (8.57%) |
|  | Non-Hispanic or Latino | 29 (82.86%) | 31 (88.57%) |
|  | Missing/Decline to Respond | 0 (0.00%) | 1 (2.86%) |
| **Marital status, n (%)** | | | |
|  | Married or partnered | 25 (71.43%) | 26 (74.29%) |
|  | Divorced, separated, or widowed | 8 (22.86%) | 3 (8.57%) |
|  | Single/never married | 2 (5.71%) | 5 (14.29%) |
|  | Missing/Decline to Respond | 0 (0.00%) | 1 (2.86%) |
| **Education, n (%)** | | | |
|  | High School Diploma | 3 (8.57%) | 2 (5.71%) |
|  | Some college/vocational school | 7 (20.00%) | 10 (28.57%) |
|  | Associate’s degree | 6 (17.14%) | 5 (14.29%) |
|  | Bachelor’s degree | 12 (34.29%) | 10 (28.57%) |
|  | Graduate degree | 7 (20.00%) | 8 (22.86%) |
| **Annual household income, n (%)** | | | |
|  | ≤ $25,000 | 2 (5.71%) | 7 (20.00%) |
|  | $25,001 - $50,000 | 12 (34.29%) | 10 (28.57%) |
|  | $50,001 - $75,000 | 7 (20.00%) | 4 (11.43%) |
|  | > $75,000 | 10 (28.57%) | 9 (25.71%) |
|  | Missing/Decline to Respond | 4 (11.43%) | 5 (14.29%) |
| Daily computer use, *n* (%) | | 25 (71.43%) | 21 (60.00%) |
| Daily internet use, *n* (%) | | 29 (82.86%) | 29 (82.86%) |
| **Years in relationship with partner, n (%)** | | | |
|  | < 10 years | 10 (28.57%) | 12 (34.29%) |
|  | 10 – 29 years | 15 (42.86%) | 15 (42.86%) |
|  | ≥ 30 years | 9 (25.71%) | 8 (22.86%) |
|  | Missing/Decline to Respond | 1 (2.86%) | 0 (0.00%) |

Note. Percentiles may not equal 100% exactly secondary to rounding error.
