# Supplementary material for: Outcomes of a Remotely Delivered Complementary and Integrative Health Partnered Intervention to Improve Chronic Pain and Posttraumatic Stress Disorder Symptoms: Randomized Controlled Trial
Source: J Med Internet Res. 2024 Oct 18;26:e57322. doi: 10.2196/57322 (PMC11530734; doi:10.2196/57322)
Supplement: Multimedia Appendix 4 [file jmir_v26i1e57322_app4.docx]

Outcome domains endorsed in interviews by unique respondent type.

| Outcome  Domain | Unique veterans referring to self | | | Unique partners referring to veteran | | | Unique partners referring to self | | |
| --- | --- | --- | --- | --- | --- | --- | --- | --- | --- |
|  | Positive | Neutral | Negative | Positive | Neutral | Negative | Positive | Neutral | Negative |
| Pain, n (%) | 12 (70.59%) | 5 (29.41%) | 0 (0.00%) | 2 (22.22%) | 7 (77.78%) | 0 (0.00%) | 4 (80.00%) | 0 (0.00%) | 1 (20.00%) |
| PTSD^a^, n (%) | 7 (53.85%) | 6 (46.15%) | 0 (0.00%) | 0 (0.00%) | 0 (0.00%) | 0 (0.00%) | 0 (0.00%) | 0 (0.00%) | 0 (0.00%) |
| Relationship, n (%) | 15 (68.18%) | 7 (31.81%) | 0 (0.00%) | 15 (100.00%) | 0 (0.00%) | 0 (0.00%) | 26 (96.00%) | 1 (4.00%) | 0 (0.00%) |
| Quality of life, n (%) | 23 (88.46%) | 3 (11.54%) | 0 (0.00%) | 9 (19.15%) | 38 (80.85%) | 0 (0.00%) | 27 (87.09%) | 4 (12.90%) | 0 (0.00%) |
| Sleep, n (%) | 5 (83.33%) | 1 (16.67%) | 0 (0.00%) | 1 (100.00%) | 0 (0.00%) | 0 (0.00%) | 10 (100.00%) | 0 (0.00%) | 0 (0.00%) |

^a^PTSD: Posttraumatic Stress Disorder
