# Supplementary material for: Outcomes of a Remotely Delivered Complementary and Integrative Health Partnered Intervention to Improve Chronic Pain and Posttraumatic Stress Disorder Symptoms: Randomized Controlled Trial
Source: J Med Internet Res. 2024 Oct 18;26:e57322. doi: 10.2196/57322 (PMC11530734; doi:10.2196/57322)
Supplement: Multimedia Appendix 5 [file jmir_v26i1e57322_app5.docx]

| Veteran and partner dyad quotes representing qualitative domains. | | | |
| --- | --- | --- | --- |
| Domain | Subdomain | Veteran Quotes | Partner Quotes |
| Biological | Pain | “[The loosening and relaxing tool] helped me loosen up from the stresses of the day and then it would lubricate my joints in the morning. Because … when I wake up in the morning, I’m very stiff. …It would help bring my pain down at night, so I could go to sleep, and then help lubricate the joints when I wake up.” | “I have chronic pain issues. So, being mindful of when I’m getting really tense and then just stopping and focusing on the areas where I’m holding a lot of that tension so that I can loosen, especially around my neck and shoulders is something that I’ve done a lot just as a strategy for dealing with pain and tightness.” |
|  |  | “Waking up my body, really helped, the reset and refresh really helped me with my pain. Definitely the loosening and relaxing. Me stretching with my pain actually helped make me feel better and gave me a somewhat better attitude.” | “Instead of taking a dose of medication that you don’t want to take, let’s try [Deep Relaxation] in the interim. If it works, then we can hold off on the medication. We tried to make it an alternative to taking pain medication...it didn’t work all the time, but the times it worked, it’s something I could sort of put into my toolbox that I can try...” |
|  |  | “[Mission Reconnect] helps but I’ve had days where regardless of what I do, my pain is still there.” | “I don’t like massage. It hurts. I don’t know why. It doesn’t take much … it’s just I’m over-sensitive …. I can’t even go to a masseuse. It just, it hurts.” |
|  | Sleep | “I would do a little routine before bed… I’d flex my calf and let it relax and feel the bed sinking me in. Then flex my chest and then flex my back, flex my biceps, flex my triceps, and then go to sleep. I feel [loosening and relaxing] helps me fall asleep easily.” | “I had time to go through [Movement into Stillness]. And that helped me to fall asleep, which was good, because I haven’t been sleeping very well.” |
|  |  | “I’m a night hawk, so I’m usually up 2:00, 3:00, 4:00 A.M. And with that, I did deep relaxation a lot… [deep relaxation] brought my thoughts where they need to be…if I would do the deep relaxation too long, I’d mess around and get a little tired.” | “I definitely feel [Mission Reconnect], gave me more tools to use at home and brought them to light more…and those nights I did tend to sleep much better, for sure.” |
|  |  | “[Massage] was always euphoric almost. I felt like I was drugged or something. It felt like my whole body was tingling. And I’d fall asleep.” | “… right before I go to bed I’ll do [Deep Relaxation]. That way I could fall asleep, and I could wake up refreshed.” |
| Psychological | PTSD Related Symptoms | “I think [Mission Reconnect] helps [PTSD symptoms] because it gives me a reason to do things. And it’s actually very easy to follow.” |  |
|  |  | “[Mission Reconnect] has kept me more in tune with what’s going on in my reality. Which is, there’s nothing out there that can really hurt me now, and nobody out there trying to kill me, and do all kinds of crazy stuff. I’m not trying to do anything crazy to anybody else.” |  |
|  |  | “I have PTSD, and I still have recurring nightmares of Vietnam, even after all this time. I’ve tried going to group therapies, and it didn’t seem to help. I’ve tried following what [Mission Reconnect] said to try and nothing seems to really help.” |  |
|  | Anxiety and Depression | “I've been diagnosed with PTSD and this month I had a lot of anxiety… so I had to go on the [Mission Reconnect] apps that can help me breathe through those things.” |  |
|  |  | “When I felt my anxiety getting up, I would go to my upstairs balcony and just sit in a chair and give myself time to get down and meditate. It’s only temporary, I can get through this...I thought [Mission Reconnect] was very good for that aspect.” |  |
|  | Relationships with others | “…she’s not talking about divorce anymore, she’s talking about, let’s go to tai chi, let’s go here.” | “I found [Mission Reconnect] was a good resource and was beneficial for my wife and I in reconnecting with each other in a more meaningful way.” |
|  |  | “[Mission Reconnect] really got me communicating better with my wife. Letting her know how I felt…which I didn’t necessarily do in the past. The main thing I got out of it was the communicating of my thoughts to her.” | “[Meditation] probably has strengthened our relationship because we do talk after we do it, and we figure out, was it helpful, or was it not helpful, how did you like that? It …strengthened our relationship, I’m sure.” |
|  |  | “I do think that [Mission Reconnect] greatly increased our relationship, with my wife. Before, it was almost like more roommates always trying to get everything done in the house and with the kids. We’re always trying to do something to make sure that everything is done and there’d be little time for us...Once we were able to say: This is gonna be the us time, that helped us come together more and be able to take that pause... We can do a date night, and go to learn how to dance, and do these other things for us. … Mission Reconnect [was] creating that intimacy without obligation, it led us to be able to dance together without that hesitation that you get with a new partner. It allowed us just to move together better and easier, and more relaxed and comfortable with each other...” | “[Mission Reconnect] Really did help with that connection between my husband and I because I didn’t realize how much he was really in pain. You could see the sorrows in his eyes, you could see the hurt and the pain in his eyes, in which I never really saw before. I was always distracted with a lot of things. And we didn’t have that good communication. It got to a point where it was hard to be in the same room. I didn’t realize how much weight my husband was carrying until I sat down in those quiet moments of reconnecting with my partner. I was like, wow, you really are in pain.” |
|  | Quality of life | “[While using centering, or the movement into stillness] My heart rate goes down. My breathing comes back down. I am not shaking anymore. I can sit there and take a few deep breaths again and say—Okay. I can do this.” | “When I get frustrated, I will go someplace and try to get into a deep meditation...I cut all sound …Everything changes after I go do maybe an hour of [deep medication] and come back, I got a whole new attitude. I’m not upset, I’m not in no confrontational mood anymore.” |
|  |  | “[Loosening and Relaxing] was probably one of the other most important things for me...it was just me relaxing, getting my breathing going. It made me aware that—Hey, you know, maybe you’re a little tight right here, let me do this to feel better.” | “Morning Gratitude was a way to connect with the day...and start new and fresh … it's a little reset to the day...” |
|  |  | “See things for what they are and not what you think they might have been or may be in the future just for what they are. My life would be a lot simpler and [MR] did help bring some of these things into my focus.” | “Centering] gave me a minute to reflect on the things that are good and that I appreciate in my life as opposed to all the bad things.” |
